# Outcomes after fixation for undisplaced femoral neck fracture compared to hemiarthroplasty for displaced femoral neck fracture among the elderly

**DOI:** 10.1186/s12891-015-0671-6

**Published:** 2015-08-19

**Authors:** Jeff Chien-Fu Lin, Wen-Miin Liang

**Affiliations:** Department of Statistics, National Taipei University, Taipei, Taiwan; Department of Orthopedic Surgery, Wan Fang Hospital, Taipei Medical University, Taipei, Taiwan; Graduate Institute of Biostatistics, China Medical University, Taichung, Taiwan; Department of Public Health, China Medical University, Taichung, Taiwan

## Abstract

**Background:**

This study compared the rates of mortality, medical complication, and reoperation after fixation surgery for displaced femoral neck fracture with those after hemiarthroplasty surgery for undisplaced femoral neck fracture using competing risk analysis in inpatients aged 60 years and above from a population database in Taiwan.

**Methods:**

We identified 13,772 subjects who underwent fixation for undisplaced cervical fracture and 13,772 matched controls who underwent hemiarthroplasty for displaced cervical fracture from 1998 to 2007, and followed them up until the end of 2009. The outcomes of patients who received internal fixation for undisplaced fracture and those of patients who received hemiarthroplasty for displaced fracture were compared.

**Results:**

The 3-month, 2-year, and 10-year mortality rates were 4.9 %, 22.1 %, and 67.1 % for fixation, and 5.6 %, 23.8 %, and 71.0 % for hemiarthroplasty, respectively. The 3-month, 2-year, and 10-year cumulative incidence rates of the first reoperation were 7.4 %, 18.1 %, and 27.7 % for fixation and 6.3 %, 12.0 %, and 22.3 % for hemiarthroplasty, respectively. The 3-month cumulative incidence rates of the first medical complication were 14.4 % for fixation and 15.4 % for hemiarthroplasty, respectively. Hemiarthroplasty had a 1.09 times (95 % CI: 1.05–1.12) higher hazard ratio for overall death than fixation. However, fixation had a 1.36 times (95 % CI: 1.29–1.43) higher subdistribution hazard ratio for first reoperation than hemiarthroplasty after adjusting for gender, age, and comorbidities.

**Conclusions:**

The short-term overall mortality and medical complication rate of fixation for undisplaced fracture were slightly lower than those of hemiarthroplasty for displaced fracture. However, the short-term cumulative incidence of first reoperation after fixation was significantly higher than that for hemiarthroplasty. Further prospective studies or clinical trials based on the competing risk model, and which include important risk factors, are necessary to quantify the adjusted effects more precisely.

## Background

Osteoporotic hip fractures, including femoral neck fractures, often cause high mortality and morbidity in elderly adults [1–32]. The general treatments for femoral neck fractures are internal fixation for undisplaced fracture and hemiarthroplasty for displaced fracture [33, 34]. However, medical complications or surgical complications after surgery often cause repeated readmissions or reoperations because of aging and fragile bones [1–22]. Complications are associated with increasing mortality and socioeconomic burden on healthcare systems and families [1–22]. Therefore, estimation of medical complications or reoperation rates should be as accurate as possible to provide a decision-making guide for clinicians and patients after fracture.

Elderly adults with hip fractures often have multiple medical comorbidities, mental, emotional or neurological disorders, functional disability, and family or social support. Such patients often can not closely follow the postoperative ambulatory program after internal fixation for undisplaced femoral neck fracture. Therefore, the postoperative complication rate is high, which raises the question as to whether undisplaced femoral neck fracture should be treated with arthroplasty to decrease the occurrence of the postoperative complication. Most previous studies for readmissions or reoperations often did not consider the competing risk of death. The cumulative complication rates are often overestimated without considering the high death rate among the elderly [35–37]. Accordingly, the present study investigated the long-term outcomes, including mortality, medical complication, and reoperation rates after fixation surgery for undisplaced femoral neck fracture and those after hemiarthroplasty surgery for displaced femoral neck fracture using competing risk analysis of data for inpatients aged 60 years and above obtained from a nationwide population database. This study also compared the outcomes of patients who received fixation for undisplaced fracture with those of patients who received hemiarthroplasty for displaced fracture.

## Methods

### Data source and subjects

The National Health Insurance Research Database (NHIRD) contains information from 1997 to the present. This information is provided yearly by Taiwan’s National Health Research Institutes. Taiwan’s National Health Insurance program was implemented in 1995 to finance healthcare for all Taiwan residents. The database covers the medical benefit claims of more than 23 million Taiwanese residents in 2011. The coverage rate was more than 99 % of the entire population in 2011. The NHIRD is composed of anonymous secondary data released to the public for research purposes. Thus, this study was exempted from a full review by the local ethics review committee.

This study selected all subjects aged 60 years or older and who were admitted to hospitals between 1 January 1998 and 31 December 2007. The subjects were identified from the database with both (i) a first discharge diagnosis code of intracapsular (femoral neck) fracture [based on the International Classification of Diseases, Ninth Revision, Clinical Modification (ICD-9-CM) codes 820, 820.0, 820.00, 820.01, 820.02, and 820.09] and (ii) medical code corresponding to surgery for internal fixation or hemiarthroplasty (based on ICD-9-CM codes 79.15, 79.35, and 81.52). The first admission day of femoral neck (cervical) fracture was defined as the index day. The exclusion criteria were inpatients with pathological fractures (ICD-9-CM codes 733.14 and 733.15) or open hip fractures (ICD-9-CM codes 820.1, 820.10, 820.11, 820.12, 820.19, and 820.9). Patients who had basicervical or intertrochanteric fractures; operation over the pelvic, femoral, or hip regions before the index day were excluded to avoid confounding effects. During the study period, all 13,772 subjects aged ≧60 years with undisplaced cervical fractures were treated with internal fixation initially, and more than 99 % of 52,276 subjects aged ≧60 years with displaced cervical fractures were treated with hemiarthroplasty. According to the guidelines of the National Health Insurance (NHI) program in Taiwan, Garden Types III and IV are classified as displaced femoral neck fracture, and Garden Types I and II are classified as undisplaced fracture. The NHIRD also contains additional information in the form of “National Insurance Codes”, which are different from ICD-9 codes, to allow the identification of undisplaced and displaced femoral neck fracture. Elderly subjects (60 years or older) with displaced femoral neck fracture are treated with hemiarthroplasty and patients with undisplaced femoral neck fracture are treated with internal fixation. The completeness and accuracy of the NHI database is guaranteed by the Ministry of Health and Welfare and the NHI Bureau of Taiwan. Because a number of covariate differences exist among the elderly with hip fracture, we used a matched cohort design to explore the outcomes of fixation for undisplaced and hemiarthroplasty for displaced cervical fracture. For each undisplaced cervical fracture with internal fixation, we randomly matched one displaced cervical fracture with hemiarthroplasty to serve as a control. Each matched control received surgery in the same calendar year, and had the same age, gender, and level of Charlson Comorbidity Index (CCI). Both cohorts were followed up until the end of the period of observation on December 31, 2009, withdrawal from the NHI program, or death.

### Outcomes of interest

This study analyzed three outcomes: (a) overall cumulative mortality; (b) cumulative incidence of the first reoperation, and (c) cumulative incidence of the first medical complication within 90 days after the index surgery. The overall survival time was defined as the duration from the index day to the death day. Subjects who survived at the end of study or were lost to follow-up were treated as censored. The first reoperation time was defined as the duration from the index day to the day on which the first postoperative unplanned reoperation occurred. The causes of the reoperations included surgical site infection, mechanical complication, loss of reduction, screw back out or cut out, skin irritation, implant failure, malunion/nonunion, second hip fracture, dislocation, and avascular necrosis of the femoral head during follow-up after the index surgery. The first medical complication time was defined as the duration from the index day to the day of the first medical complication within 90 days after the index surgery that required longer hospital stay or readmission to the hospital for treatment. The 90-day medical complications included stroke, acute myocardial infarction, pulmonary embolism, acute renal failure, and acute respiratory failure that occurred within 90 days after the index surgery. The comorbidities of each subject were retrieved before or at the time of the index day on the basis of the Charlson comorbidity index (CCI) [38].

### Statistical analysis

We estimated the survival rate on the basis of the Kaplan–Meier (KM) method to analyze the overall survival. We explored the effects of risk factors on survival using the log-rank test and multiple Cox’s proportional hazards model. Osteoporotic hip fracture causes excess mortality and thus there were no data for complications in subjects who died. That is to say, for subjects who had died, there were no complications after the date of death. In this situation, death is referred to as a “competing risk” for the outcomes (medical complications or reoperations) of interest. A competing risk of death is an alternative outcome that is equally or more important than the primary outcome and alters the probability of the outcome of interest. Statistical approaches, such as KM estimator and Cox’s proportional hazards model, are typically applied to describe all-cause mortality rather than the incident disease or complication. When KM estimates are used to describe outcomes other than all-cause mortality in the presence of a significant and related competing risk of death, the KM estimates lead to biased results because the proportion of death is large and increases with follow-up time. An alternative statistical approach has been developed to account for the presence of competing risks and is termed “competing risk analysis”. In our study, subjects had a markedly high number of comorbidities, so the competing risk of death was especially high compared to the risk of complications. It is important to adequately account for the competing risk of death in the analyses. Therefore, we estimated the cumulative incidence of the first medical complication or the first reoperation according to the cumulative incidence function of competing risk analysis to analyze the complication-free time [35–37, 39, 40]. We explored the effects of risk factors on complication-free time using Gray’s test on the basis of the Fine and Gray model, i.e., the subdistribution proportional hazards model. The risk factors include age, gender, operation type, and the CCI. We compared the outcomes between fixation for undisplaced and hemiarthroplasty for displaced femoral neck fracture based on the matched cohort design. All data management and analysis were performed using the SAS system (version 9.3; SAS Institute, Cary, NC) and R 3.0.0 [available at http://www.R-project.org/, R Development Core Team (2013), R: A language and environment for statistical computing. R Foundation for Statistical Computing, Vienna, Austria, especially the R libraries survival, cmprsk, and mstate].

## Results

**Fig. 1:**
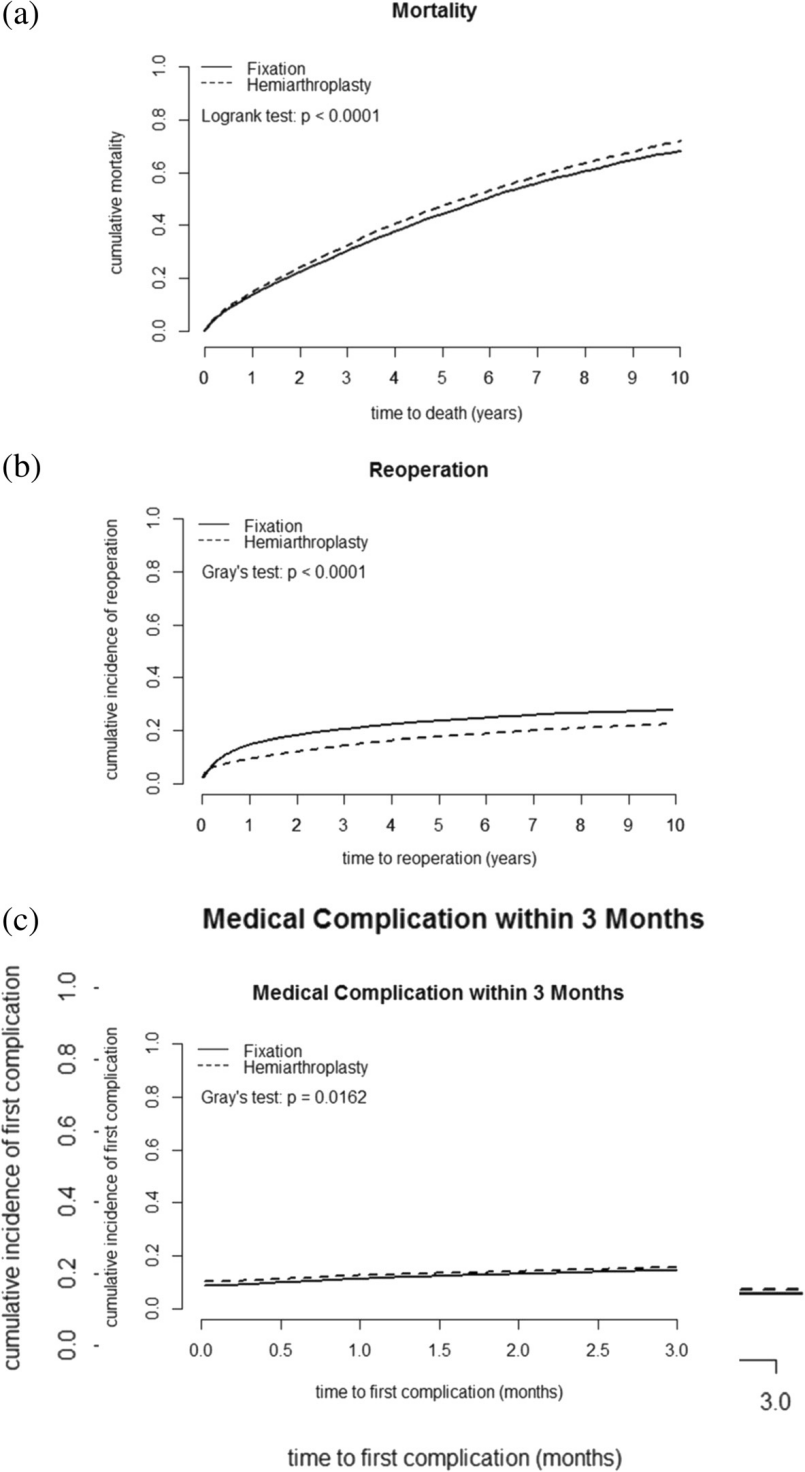
Ten-year cumulative curves of (**a**) mortality stratified by treatments, (**b**) the first reoperation stratified by treatments, (**c**) three-month cumulative curves of first medical complication stratified by treatments

Between 1998 and 2007, we identified 13,772 subjects with internal fixation for undisplaced cervical fracture and 13,772 matched controls with hemiarthroplasty for displaced cervical fracture (Table 1). The 1-month, 3-month, 6-month, 1-year, 2-year, 5-year, and 10-year mortality rates and cumulative incidence rates of the first medical complications and the first reoperations are shown for the fixation and hemiarthroplasty cohorts, respectively, in Table 2 and Fig. 1. The median survival times were 5.96 (95 % CI: 5.83–6.12) years for the undisplaced fracture with fixation and 5.53 (95 % CI: 5.39–5.70) years for the displaced fracture with hemiarthroplasty. Hemiarthroplasty for displaced fracture had a higher mortality rate than that of fixation for undisplaced fracture. However, fixation for undisplaced fracture had higher cumulative incidence rates for the first reoperation than those of hemiarthroplasty for undisplaced fracture (Fig. 1).

**Table 1:** Baseline characteristics of femoral neck fractures stratified by fracture type

		Undisplaced fracture	Displaced fracture	
		Fixation	Hemiarthroplasty	
		*N* = 13772 (50.0 %)	*N* = 13772 (50.0 %)	*p*-value
Age (years), Mean ± SD		76 ± 7.9	76 ± 7.9	0.99
Gender	Female	8356 (60.7 %)	8356 (60.7 %)	0.99
	Male	5416 (39.3 %)	5416 (39.3 %)	
CCI^a^	0	6192 (45.0 %)	6192 (45.0 %)	0.99
	1	3448 (25.0 %)	3448 (25.0 %)	
	2	2079 (15.1 %)	2079 (15.1 %)	
	3	1123 (8.2 %)	1123 (8.2 %)	
	≧4	930 (6.8 %)	930 (6.8 %)	
Hyptertension	No	8039 (58.4 %)	7718 (56.0 %)	<0.01
	Yes	5733 (41.6 %)	6054 (44.0 %)	
Diabetes Mellitus	No	10986 (79.8 %)	10915 (79.3 %)	0.30
	Yes	2786 (20.2 %)	2857 (20.7 %)	
Heart Disease	No	11571 (84.0 %)	11579 (84.1 %)	0.91
	Yes	2201 (16.0 %)	2193 (15.9 %)	
COPD	No	11460 (83.2 %)	11655 (84.6 %)	<0.01
	Yes	2312 (16.8 %)	2117 (15.4 %)	
Cerebrovascular	No	11274 (81.9 %)	11199 (81.3 %)	0.25
	Yes	2498 (18.1 %)	2573 (18.7 %)	
Chronic Liver Disease	No	13153 (95.5 %)	13144 (95.4 %)	0.82
	Yes	619 (4.5 %)	628 (4.6 %)	
Chronic Renal Disease	No	13142 (95.4 %)	13136 (95.4 %)	0.89
	Yes	630 (4.6 %)	636 (4.6 %)	

^a^Charlson comorbidity index

**Table 2:** Cumulative mortality rates and cumulative incidence of the first reoperation or medical complication after surgery for femoral neck fracture stratified by type of fracture

			Cumulative Incidence			
	Mortality		Reoperation		Medical Complication	
	Undisplaced	Displaced	Undisplaced	Displaced	Undisplaced	Displaced
Time	Fixation	Hemiarthroplasty	Fixation	Hemiarthroplasty	Fixation	Hemiarthroplasty
1-month	1.7 %	1.9 %	3.5 %	4.3 %	11.2 %	12.5 %
3-month	4.9 %	5.6 %	7.4 %	6.3 %	14.4 %	15.4 %
6-month	8.3 %	9.1 %	10.9 %	7.4 %		
1-year	13.4 %	14.6 %	14.6 %	9.2 %		
2-year	22.1 %	23.8 %	18.1 %	12.0 %		
5-year	43.7 %	46.9 %	23.6 %	17.5 %		
10-year	67.1 %	71.0 %	27.7 %	22.3 %

We explored the effects of risk factors on survival using multivariate survival analysis. Male gender, older age, hemiarthroplasty, and higher Charlson comorbidity index were significant risk factors for mortality. Males had a 1.42 times (95 % CI: 1.38–1.47) higher hazard ratio (HR) of overall death than females. For each one-year increase in age, the HR increased by 1.06 times (95 % CI: 1.06–1.07). Hemiarthroplasty had a 1.09 times (95 % CI: 1.05–1.12) higher HR of overall death than fixation. Patients with one, two, three, and four or more CCI had higher HRs of overall death compared with those with no comorbidity (HR: 1.53, 95 % CI: 1.47–1.59; HR: 2.01, 95 % CI: 1.91–2.10; HR: 2.34, 95 % CI: 2.21–2.48; and HR: 3.53, 95 % CI: 3.33–3.75, respectively) (Table 3).

**Table 3:** (a) Hazard ratios of the risk factors associated with death from cause-specific hazard analysis based on Cox’s model; (b) sub-distribution hazard ratios of the risk factors associated with the first reoperation, and (c) the medical complication based on Fine and Gray’s (sub-distribution hazard) model from competing risk analysis

		(a) Death				(b) Reoperation				(c) Medical complication			
		HR	95 % Cl		*p*-value	Sub-HR	95 % Cl		*p*-value	Sub-HR	95 % Cl		*p*-value
Age	Years	1.063 (1.061, 1.065)			< .0001	0.990 (0.987, 0.994)			< .0001	1.041 (1.037, 1.045)			< .0001
Operation	Fixation	1(REF.)				1(REF.)				1(REF.)			
	Hemiarthroplasty	1.085 (1.051, 1.121)			< .0001	0.738 (0.701, 0.776)			< .0001	1.080 (1.019, 1.145)			0.009
Gender	Female	1(REF.)				1(REF.)				1(REF.)			
	Male	1.423 (1.378, 1.471)			< .0001	1.019 (0.968, 1.073)			0.470	1.749 (1.649, 1.854)			< .0001
CCI^a^	0	1(REF.)				1(REF.)				1(REF.)			
	1	1.529 (1.467, 1.594)			< .0001	1.000 (0.940, 1.064)			0.997	4.536 (4.114,5.001)			< .0001
	2	2.005 (1.912, 2.102)			< .0001	0.927 (0.858, 1.001)			0.053	5.607 (5.065, 6.207)			< .0001
	3	2.339 (2.208, 2.477)			< .0001	0.875 (0.791, 0.969)			0.010	6.416 (5.739, 7.173)			< .0001
	≧4	3.534 (3.330, 3.750)			< .0001	0.913 (0.818, 1.020)			0.107	7.818 (7.000, 8.733)			< .0001

^a^Charlson comorbidity index

We explored the effects of risk factors on the time to the first reoperation with multivariate competing risk analysis. Age, fixation, and higher CCI were significant risk factors for reoperation. After controlling for age, gender, and comorbidities, hemiarthroplasty had a 0.74 times (95 % CI: 0.70–0.78) lower sub-HR for reoperation than fixation. We also explored the effects of risk factors on the time to the first medical complication. Age, male gender, hemiarthroplasty, and higher CCI were significant risk factors for medical complication within 90 days after surgery. After controlling for age, gender, and comorbidities, hemiarthroplasty had a 1.08 times (95 % CI: 1.02–1.15) higher sub-HR for medical complication than fixation (Table 3).

Subjects often had multiple medical complications and reoperations during follow-up. Among the subjects with fixation who survived within 90 days after the index surgery, 1989 had at least one medical complication. Most of medical complications were acute pulmonary disorders, such as acute exacerbation of chronic obstructive lung disease, pneumonia, acute respiratory failure, and stroke. Two thousand six hundred and ninety-one subjects with fixation had at least one reoperation within 2 years after the index surgery. Most reoperations were caused by infection (12.0 %), mechanical complication (32.4 %, including loss reduction, screw back out or cut out, skin irritation, and implant failure) and avascular necrosis of the femoral head (19.18 %). The percentage of implant removal was 42.55 %, resulting in a conversion to/revision arthroplasty rate of 37.9 % among 2691 study patients (Table 4). Once subjects had the first medical complication, 22.9 % of subjects with fixation for undisplaced cervical fracture died within 3 months compared to 21.9 % of the matched controls with hemiarthroplasty for displaced cervical fracture. Furthermore, once subjects had the first reoperation, 14.7 % of subjects with fixation died within 1 year compared to 21.6 % of the controls (data not shown in the tables).

**Table 4:** Causes of reoperations or medical complications after surgery for hip fracture, stratified by fracture type

(1) Reoperation														
	1-Month		3-Month		6-Month		1-year		2-year		5-Year		10-Year	
Fracture type^a^	Un-dis	Dis	Un-dis	Dis	Un-dis	Dis	Un-dis	Dis	Un-dis	Dis	Un-dis	Dis	Un-dis	Dis
Operation type	Fixation	Bipolar^a^	Fixation	Bipolar	Fixation	Bipolar	Fixation	Bipolar	Fixation	Bipolar	Fixation	Bipolar	Fixation	Bipolar
n^b^	978	594	1466	860	1901	1021	2326	1268	2691	1650	3172	2311	3405	2615
Conversion to/revision arthroplasty	17.28%^c^	15.66 %	38.20 %	19.19 %	48.97 %	24.88 %	56.32 %	32.10 %	60.42 %	37.82 %	63.71 %	42.62 %	63.41 %	44.09 %
Removal of implant	15.03 %	10.61 %	29.81 %	11.63 %	36.61 %	12.73 %	41.19 %	12.15 %	42.55 %	12.06 %	41.49 %	10.39 %	40.03 %	10.02 %
Infection	9.30 %	46.80 %	10.91 %	43.14 %	10.73 %	40.55 %	10.83 %	35.73 %	12.00 %	31.15 %	12.89 %	26.27 %	13.33 %	25.05 %
Mechanical complication^d^	21.98 %	22.90 %	34.17 %	24.65 %	35.56 %	24.29 %	33.53 %	24.61 %	32.40 %	24.42 %	30.67 %	22.89 %	29.63 %	22.29 %
Dislocation	NA	23.57 %	NA	25.81 %	NA	23.11 %	NA	19.87 %	NA	16.42 %	NA	12.81 %	NA	11.66 %
Avascular necrosis	52.76 %	NA	35.20 %	NA	27.14 %	NA	22.18 %	NA	19.18 %	NA	16.27 %	NA	15.15 %	NA
Same site second hip fracture	1.33 %	3.87 %	3.07 %	5.58 %	4.10 %	6.37 %	4.43 %	7.89 %	5.20 %	8.91 %	6.46 %	10.69 %	6.93 %	11.17 %
Malunion	0.92 %	NA	0.75 %	NA	0.63 %	NA	0.77 %	NA	0.78 %	NA	0.79 %	NA	0.79 %	NA
Nonunion	3.07 %	NA	8.73 %	NA	14.68 %	NA	19.60 %	NA	19.55 %	NA	18.06 %	NA	16.98 %	NA
(2) Medical Complication														
					1-Month		3-Month							
Fracture type^a^					Un-dis	Dis	Un-dis	Dis						
Operation Type					Fixation	Bipolar	Fixation	Bipolar						
n^b^					1543	1726	1989	2126						
Stroke					12.70%^c^	11.88 %	14.43 %	12.98 %						
Acute myocardial infraction					3.18 %	2.61 %	3.42 %	3.15 %						
Pulmonary embolism					1.17 %	1.85 %	1.21 %	1.60 %						
Deep vein thrombosis					1.04 %	1.04 %	1.31 %	1.22 %						
Acute renal failure					5.12 %	6.55 %	7.04 %	8.42 %						
Acute respiratory failure					17.17 %	16.74 %	21.82 %	20.93 %						
pneumonia					25.15 %	25.38 %	30.87 %	32.13 %						
Exacerbation of COPD					59.17 %	59.33 %	53.44 %	54.66 %						

*NA* Not available

^a^Fracture type : Un-Dis = Undisplaced; Dis = Displaced femoral neck fracture; Bipolar = Hemiarthroplasty

^b^ *n* = The number of subjects who had at least one readmission or reoperation

^c^% = Percentage of subjects who had a certain cause of complication among the total number of subjects who had at least one complication or reoperation. Subjects might have had more than one readmission or reoperation due to multiple causes

^d^Mechanical complication included loss reduction, screw back out or cut out, skin irritation, and implant failure

## Discussion

In this population study, mortality, medical complication, and reoperation rates were analyzed simultaneously after surgery for femoral neck fracture among elderly adults. For both treatments, the cumulative incidence of the first reoperation rapidly increased within 2 years after surgery and slowed down later (Fig. 1). Several individual and meta-analysis studies reported that hip fractures affect short-term but not long-term mortality [28, 29, 41–44]. It is unclear whether undisplaced femoral neck fracture with arthroplasty can decrease long-term postoperative complication rates. We found that the short-term overall mortality and medical complication rates in patients who received undisplaced femoral neck fracture treated with fixation was approximately 1 % lower than that of patients whose displaced femoral neck fracture was treated with hemiarthroplasty. Multiple factors contributed to the higher short-term mortality that was associated with hemiarthroplasty, including type of fracture, greater invasiveness of surgery and more technically demanding, longer surgical time, more blood loss, bone cement implantation syndrome, thromboembolism, and postoperative infection. The difference in mortaliy may be atttributed to fracture type based on our retrospective cohort database. Few clinical studies have directly compared 4 groups: undisplaced fracture with internal fixation, undisplaced fracture with hemiarthroplasty, displaced fracture with internal fixation, and displaced fracture with hemiarthroplasty. Few clinical trials have directly compared fixation and hemiarthroplasty for undisplaced femoral neck fracture. We suggest that prospective studies and clinical trials are necessary to directly compare the outcomes between fixation and hemiarthroplasty for undisplaced fracture. In addition, the one-year cumulative incidence of the first reoperation after fixation for undisplaced fracture was 4 %-5 % higher than that of hemiarthroplasty for displaced fracture, which was significantly different (Tables 2 and 3). However, the long-term overall mortality associated with fixation for undisplaced femoral neck fracture was significantly lower than that of hemiarthroplasty for displaced femoral neck fracture. Moreover, the long-term cumulative incidence of the first reoperation after fixation was significantly higher than that for hemiarthroplasty.

Some observational studies obtained different results after comparing fixation for undisplaced fracture and arthroplasty for displaced fracture [1, 3, 5, 11, 22]. Teixeira et al. used competing risk analysis for 1-year cumulative incidence of readmission after surgery for hip fracture [22]. They found that among 5709 patients, 32 % had at least one readmission, that nearly 80 % of readmissions occurred within 3 months after discharge, and that 53 % of readmissions were fracture-related [22]. Sikand, Wenn, and Moran evaluated outcomes for 139 undisplaced femoral neck fractures (110 for fixation and 29 for hemiarthroplasty). They found that the 1-year mortality rate was 16 % for fixation, which was significantly lower than that (38 %) for hemiarthroplasty. They also found that the 1-year reoperation rate was 7.2 % for fixation, which was significantly higher than that (3 %) for hemiarthroplasty [5]. Radcliff et al. evaluated a national sample of 5683 male elderly veterans after surgery for hip fractures. They found that the overall 30-day mortality rate, readmission rate, and reoperation rates were 8 %, 7 %, and 21 %, respectively. They did not find any significant differences among the types of various surgical operations [11]. In the present study, the results of competing risk analysis showed that the 1-year cumulative incidence rates of the first reoperation of fixation for undisplaced fracture and hemiarthroplasty for displaced fracture were 14.6 % and 9.2 %, respectively. Directly comparing the results may be difficult because of the different definitions of unplanned readmission or reoperation criteria.

Several prospective studies or clinical trials reported mortality and reoperation rates simultaneously after fixation or hemiarthroplasty for displaced fractures [15, 23–27, 45–47]. Most of these studies did not find significant differences in mortality. These studies also reported that fixation had a significantly higher reoperation rate than that of hemiarthroplasty [15, 23–27, 45–47]. Possibly, these trials did not have a large enough sample size or a sufficiently long follow-up time to achieve enough statistical power to detect a difference in mortality. In our database, all undisplaced femoral neck fractures were treated with fixation, and more than 99 % of displaced femoral neck fractures were treated with hemiarthroplasty for subjects aged 60 years and above during the study period. Our study included only subjects aged 60 years and older with femoral neck fracture. We did not have any subjects who underwent hemiarthroplasty for undisplaced fracture. Therefore, the fixation group included subjects with undisplaced fracture, and the hemiarthroplasty group included subjects with displaced fracture based on a matched cohort design.

Rogmark found that 40 % of 224 subjects who underwent fixation for undisplaced femoral neck fracture had persistent mild or severe walking pain after a mean follow-up time of 32 months [14]. Hui et al. also suggested that internal fixation is not the best treatment for extremely elderly patients with minimally displaced or impacted intracapsular fractures of the femoral neck [32]. However, after fixation failure, whether or not elective conversion to arthroplasty would have better or equivalent results compared with primary hemiarthroplasty remains controversial. Gjertsen et al. analyzed the Norwegian Hip Fracture Register Data which includes 4468 patients with fixation for undisplaced femoral neck fractures and 6900 patients with hemiarthroplasty for displaced fractures [16]. Gjertsen et al. found that the 1-year implant survival rate was 89 % after fixation for undisplaced fractures, and 97 % after hemiarthroplasty for displaced fractures [16]. We found that most medical complications were acute pulmonary disorders. These results were similar to those of other studies that reported the medical complication rates within 3 months varied between 10 %-20 % and most were respiratory disorders [4, 10–13, 17, 18, 22].

Hu et al. conducted a meta-analysis and estimated that males had a 1.70 times higher HR of overall death than that of females [30]. In the present study, males had a 1.42 times higher HR of overall death than females. Hu et al. also estimated that the yearly HR for age was 1.05. In the present study, the yearly HR for age was 1.06. Our result lies around the middle of the range reported in various studies conducted in different regions [30]. We found that higher CCI had a higher risk for mortality, medical complication, and reoperation. A consensus about which comorbidities should be measured and how those comorbidities should be placed into a statistical model is still lacking.

Most previous studies of femoral neck fracture used Kaplan–Meier estimates to report readmission or reoperation rates as the primary outcomes [1–21]. However, osteoporotic hip fracture causes excess mortality. Therefore, subjects who died could not have any complications. The cumulative medical complication and reoperation rates would be overestimated based on the traditional Kaplan–Meier estimates. The Kaplan–Meier estimates are biased because the proportion of death is large and increases with follow-up time. We used cumulative incidence function based on a competing risk analysis to obtain a more valid estimate of the risk for readmission or reoperation [35–37, 39, 40]. Our result implies that subjects treated with fixation have a lower all-cause hazard for death. Therefore, subjects treated with fixation stayed longer during follow-up and were thus exposed to a greater hazard for complications. Consequently, more subjects treated with internal fixation had complications than subjects with hemiarthroplasty.

### Limitations

This study had some limitations. Unlike data in a hip fracture registry, the NHRI database does not record all clinical information. Selection biases may have existed, and caution must be taken in extrapolating our results. Subjects with femoral neck fracture as defined here were aged 60 years and older and were followed up for various durations (2 years to 12 years). Therefore, some unknown confounding factors might have existed or changed during follow-up. Although we conducted a matched cohort design and multivariate analysis to examine risk factors, many risk factors, such as time to surgery, pre-operative joint function/condition, smoking status, body mass index, bone mineral density, lifestyle factors, comorbidity severity, and quality of life, were not available for adjustment.

## Conclusions

Between 1998 and 2007, the rates of short-term mortality and medical complications of patients with undisplaced femoral neck fracture treated with fixation were slightly lower than those of patients with displaced femoral neck fracture treated with hemiarthroplasty. In addition, the one-year cumulative incidence of the first reoperation after fixation for undisplaced fracture was significantly higher than that of hemiarthroplasty for displaced fracture. The long-term overall mortality associated with fixation for undisplaced femoral neck fracture in Taiwan was significantly lower than that of hemiarthroplasty for displaced femoral neck fracture. However, the long-term cumulative incidence of the first reoperation after fixation was significantly higher than that for hemiarthroplasty. Further prospective studies or clinical trials based on the competing risk model, and which include important risk factors, are necessary to quantify the adjusted effects more precisely.
